# Seronegative cutaneous rheumatoid nodules with positive response to tofacitinib

**DOI:** 10.1016/j.jdcr.2024.05.033

**Published:** 2024-06-10

**Authors:** Matthew Helm, Calista Long, Galen Foulke

**Affiliations:** a Department of Dermatology, Penn State Milton S. Hershey Medical Center, Hershey, Pennsylvania; b Penn State College of Medicine, Hershey, Pennsylvania; c Department of Public Health Sciences, Penn State Milton S. Hershey Medical Center, Hershey, Pennsylvania

**Keywords:** rheumatoid nodules, seronegative rheumatoid arthritis, tofacitinib

## Introduction

Seronegative rheumatoid arthritis (RA) has long been recognized as a phenotype of RA without the presence of rheumatoid factor or antibodies to citrullinated protein antigens. Some studies may indicate that seronegative patients present with more severe clinical manifestations at onset; however, they seem to have a better prognosis.^1^ Although there have been advancements in managing seropositive RA resulting in improved prognosis, there have not been significant advancement in the treatment of its seronegative counterpart.^2^ Thus, prognosis of seronegative RA has remained relatively constant.

General treatment guidelines for RA include nonsteroidal anti-inflammatory drugs, glucocorticoids, disease-modifying antirheumatic drugs, and biologics. Tofacitinib, a Janus kinase (JAK) inhibitor that was Food and Drug Administration approved for RA in 2012, has been shown to be relatively well tolerated.^3^ Tofacitinib has been used in various granulomatous diseases with success.^4^ Most notably, there have been cases of lung rheumatoid nodules that have been successfully treated with tofacitinib.^5^^,^^6^ Here, we describe a patient case of seronegative cutaneous rheumatoid nodules with positive response to tofacitinib.

## Case report

**Fig 1:**
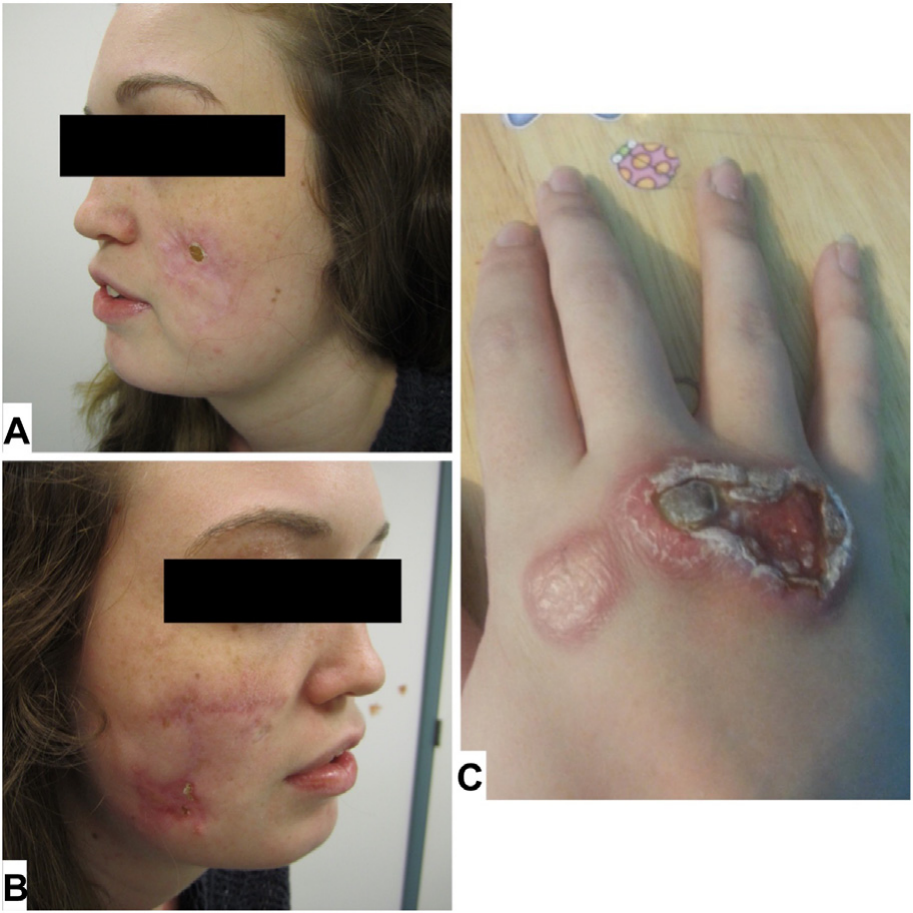
Open nodules of (**A**) the left cheek, (**B**) right cheek, and over the metacarpophalangeal joints of (**C**) the right hand before treatment with tofacitinib.

A 29-year-old woman with history of seronegative RA presented with severe pain and morning stiffness particularly of the wrists and ankles thus limiting her activities of daily living. Her symptoms began at age 8, and both her father and sister are similarly affected. Her medication trials include intralesional steroid injections, methotrexate, anakinra, infliximab, sulfasalazine, rituximab, abatacept, systemic corticosteroids, and hydroxychloroquine with some relief. Examination showed chronic nodules of the arms and legs and ulcerated nodules and plaques over the metacarpophalangeal joints of the right hand and bilateral cheeks (Fig 1). Moderate-to-severe synovitis was noted in the wrists and ankles bilaterally.

**Fig 2:**
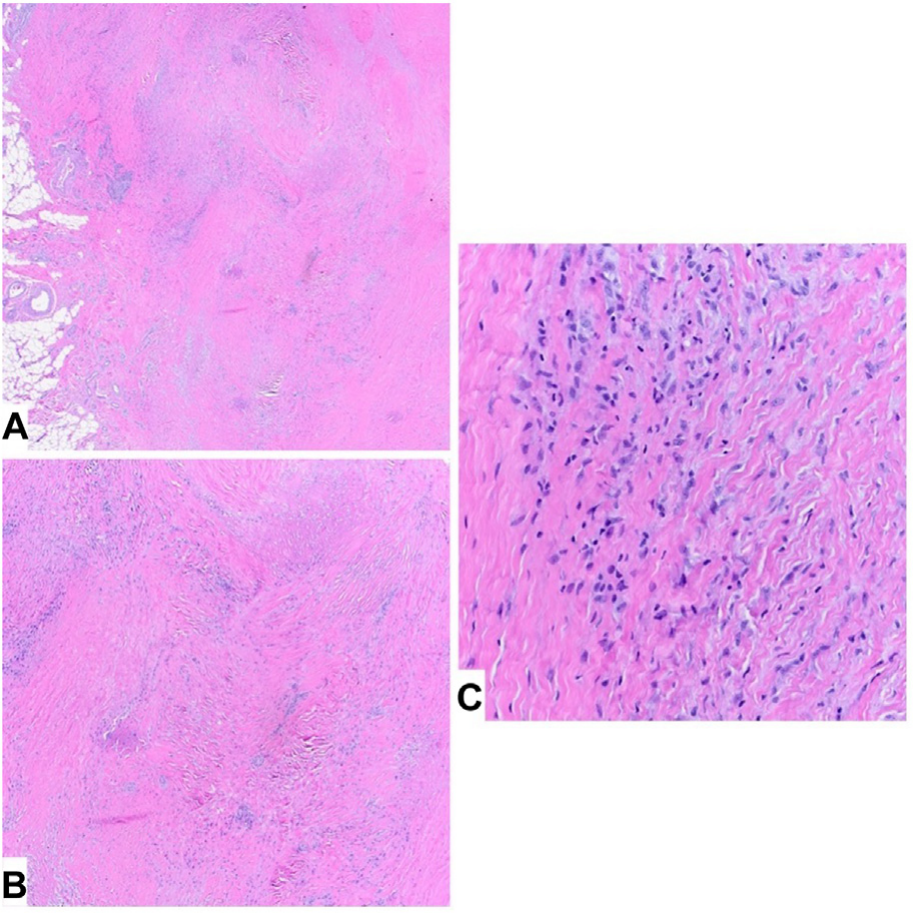
A, Palisading granulomas with central area showing fibrinoid necrosis. **B**, Palisading granulomas surrounding fibrin, with necrobiosis. **C**, Granulomatous inflammation surrounding fibrin admixed with plasma cells. (Original magnifications: **A**, ×2; **B**, ×4; **C**, ×20.)

Complete blood count, comprehensive metabolic panel, antinuclear antibody were within normal limits. C-reactive protein and erythrocyte sedimentation rate were elevated to 1.23 and 53, respectively. She was negative for Lyme disease. Rheumatoid factor was <14 and anticyclic citrullinated peptide was <20. Imaging showed soft tissue swelling and joint effusions in the knee and ankle. Biopsy showed fibrinoid necrobiotic granulomata with plasma cells, consistent with rheumatoid nodules (Fig 2).

**Fig 3:**
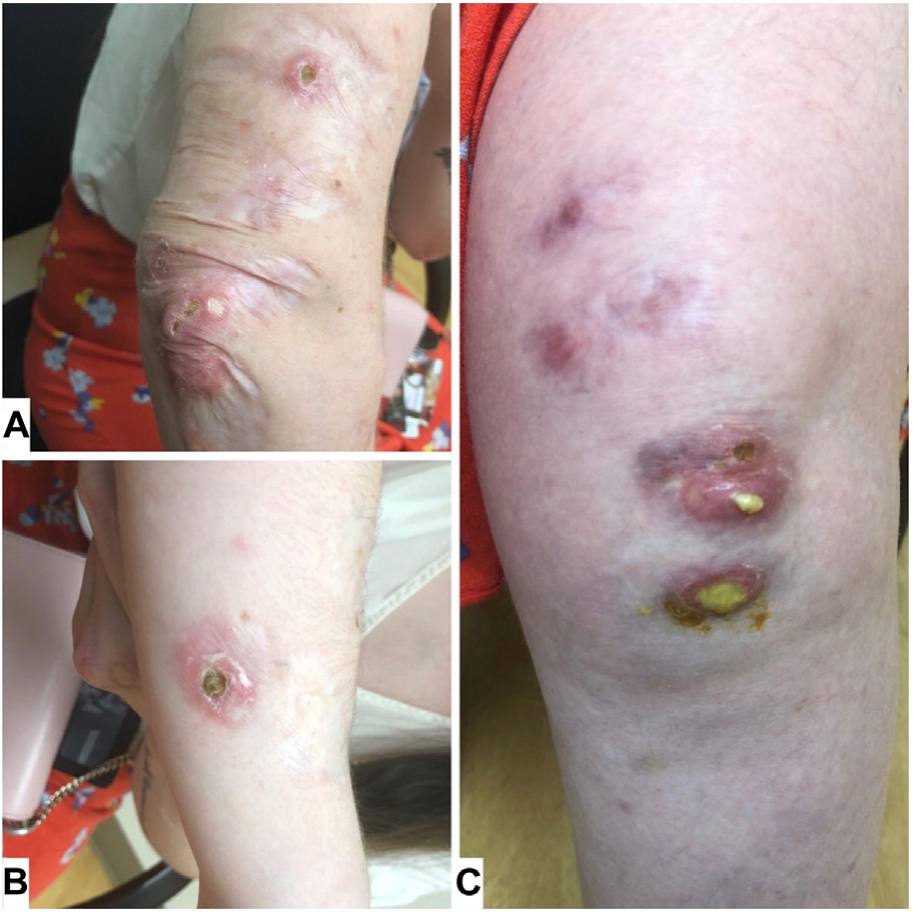
Open pink nodules of (**A**) the right elbow and (**B**) right forearm, and actively draining nodules of (**C**) the left knee with thick yellowish discharge before treatment with tofacitinib.

Because of the severity of her joint pain, and resistance to multiple other treatments, she was started on a trial of tofacitinib 5 mg twice daily and hydroxychloroquine. With treatment, the patient had improvement in her chronic rheumatoid nodules with resolution of active skin lesions. Because of a change in her insurance, the patient had to stop tofacitinib, resulting in recurrence of her skin lesions. Eight months after discontinuing the medication she was noted to have numerous atrophic scars, 3 eroded nodules with crust on the right elbow, nondraining nodules of the right knee, and 2 actively draining pink nodules of left knee with thick yellowish discharge (Fig 3).

**Fig 4:**
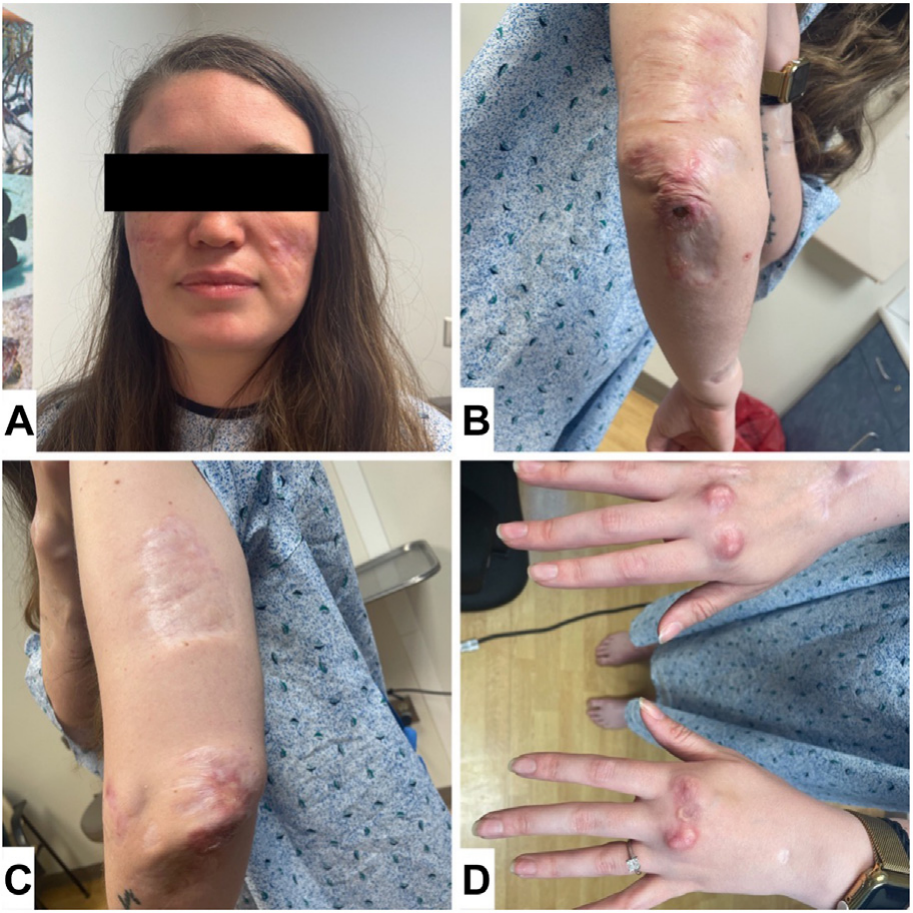
Scarred and atrophic plaques and nodules of (**A**) the face, (**B**, **C**) bilateral elbows, and (**D**) metacarpophalangeal joints after treatment with tofacitinib.

Since restarting tofacitinib, the patient was noted to have improvements in chronic rheumatoid nodules as well as a reduction in flares and open ulcers. Skin examination showed numerous scarred and atrophic plaques and nodules over the cheeks, lateral aspect of the arms, elbows, forearms, hands with emphasis at extensor joints, knees, ankles, and shins (Fig 4). Additionally, ulcers were noted to be less painful and remain open for a shorter duration of time. Her ability to ambulate and perform activities of daily living was noted to improve as well.

## Discussion

Tofacitinib is a JAK inhibitor that suppresses the phosphorylation and activation of the JAK-signal transducer and activator of transcription (STAT) signaling pathway, reducing the production of proinflammatory cytokines that regulate the immune response.^3^ It was approved in the United States in November 2012 and is indicated for the treatment of moderate-to-severe active RA in adult patients who do not respond adequately or are intolerant to 1 or more disease-modifying antirheumatic drugs, either biologic or conventional.^3^^,^^7^

Tofacitinib has recently demonstrated positive response in cutaneous conditions such as cutaneous sarcoidosis and granuloma annulare.^4^ Tofacitinib has also been noted to have a positive effect on pulmonary rheumatoid nodules in several case reports. One report described symptomatic lung rheumatoid nodules refractory to abatacept and intravenous cyclophosphamide that improved with tofacitinib treatment.^6^ Another describes a case of active RA with interstitial lung disease and a large inflammatory lung nodule that was improved with tofacitinib.^5^

Several cytokines involved in the pathogenesis of RA, such as interleukin-6 (IL-6), interferons, granulocyte-macrophage colony-stimulating factor and common gamma chain cytokine family, act through JAK-STAT pathway.^8^ In RA, STAT3 is constitutively phosphorylated in circulating T cells and monocytes suggesting hyperactivation of the IL-6–STAT3 axis. IL-6 signals through JAK1 and JAK2/Tyrosine kinase 2 and common gamma chain cytokines through JAK1 and JAK3.^8^ Interferon gamma plays a prominent role in inflammatory polarization of macrophages and granuloma formation, whereas IL-6 serves as a reinforcing cytokine, perpetuating granulomatous inflammation in the presence of pathogenic antigens.^9^ Tofacitinib, which inhibits JAK1, JAK3, and to a slightly lesser extent JAK2, therefore may function to reduce or inhibit inflammatory pathways that lead to rheumatoid nodule formation.

Our patient case demonstrates cutaneous rheumatoid nodules with positive response to tofacitinib. Tofacitinib should be further studied as a potential therapy for patients with cutaneous rheumatoid nodules.

## Conflicts of interest

None disclosed.

## References

[1] Paalanen K., Puolakka K., Nikiphorou E., Hannonen P., Sokka T. (2021). Is seronegative rheumatoid arthritis true rheumatoid arthritis? A nationwide cohort study. Rheumatol (Oxf Engl).

[2] De Stefano L., D’Onofrio B., Gandolfo S. (2023). Seronegative rheumatoid arthritis: one year in review 2023. Clin Exp Rheumatol.

[3] Álvaro-Gracia J.M., García-Llorente J.F., Valderrama M., Gomez S., Montoro M. (2021). Update on the safety profile of tofacitinib in rheumatoid arthritis from clinical trials to real-world studies: a narrative review. Rheumatol Ther.

[4] Rosenbach M. (2020). Janus kinase inhibitors offer promise for a new era of targeted treatment for granulomatous disorders. J Am Acad Dermatol.

[5] Her M., Park J., Lee S.G. (2024). A large pulmonary nodule in a rheumatoid arthritis patient treated with tofacitinib. Int J Rheum Dis.

[6] Kondo M., Murakawa Y., Honda M. (2021). A case of rheumatoid arthritis with multiple lung rheumatoid nodules successfully treated with tofacitinib. Mod Rheumatol Case Rep.

[7] US Food and Drug Administration (2017). XELJANZ(R) (tofacitinib) tablets/XELJANZ(R) XR (tofacitinib) extended release tablets. prescribing information. https://www.accessdata.fda.gov.

[8] Palmroth M., Kuuliala K., Peltomaa R. (2021). Tofacitinib suppresses several JAK-STAT pathways in rheumatoid arthritis *in vivo* and baseline signaling profile associates with treatment response. Front Immunol.

[9] Wang A., Singh K., Ibrahim W., King B., Damsky W. (2020). The promise of JAK inhibitors for treatment of sarcoidosis and other inflammatory disorders with macrophage activation: a review of the literature. Yale J Biol Med.

